# A protocol for the evaluation of the PneumoWave biosensor in supported accommodation settings: A study on feasibility and acceptability (RESCU-2)

**DOI:** 10.1371/journal.pone.0326482

**Published:** 2025-07-07

**Authors:** Basak Tas, Lewis Beer, Elizabeth Appiah-Kusi, Will Lawn, Iona Belov, Polly Radcliffe, Andrew Radley, Bruce Henderson, Osian Meredith, Catriona Cowan, Stuart Kelly, Holly Maxwell Pringle, Alex Adam, John Strang, John F. Dillon

**Affiliations:** 1 National Addiction Centre, Institute of Psychiatry, Psychology & Neuroscience (IoPPN), Addictions Department, King’s College London, London, United Kingdom; 2 School of Medicine, University of Dundee, Dundee, Scotland, United Kingdom; 3 South London & Maudsley NHS Foundation Trust, London, United Kingdom; 4 Department of Psychology, Institute of Psychiatry, Psychology & Neuroscience, King’s College London, London, United Kingdom; 5 PneumoWave Ltd, Maxim 3, Maxim Office Park, Parklands Avenue, Eurocentral, United Kingdom; PLOS: Public Library of Science, UNITED KINGDOM OF GREAT BRITAIN AND NORTHERN IRELAND

## Abstract

**Background:**

People who overdose on opioids when they are alone or unmonitored are at heightened risk of death as other people do not know they should provide an emergency response. Wearable technology provides an opportunity to continuously measure respiratory function and ultimately send an alert if respiratory depression occurs.

**Objective:**

This study evaluates the feasibility and acceptability of PneumoWave DC in UK homeless hostels or supported accommodation settings (equivalent to Housing First in the USA) for individuals at high risk of opioid overdose. The PneumoWave system consists of a wearable biosensor that is affixed to the chest and records chest motion and which, in future, could potentially provide early detection of respiratory depression and trigger overdose response.

**Methods:**

RESCU-2 is a non-randomised, observational trial conducted in supported accommodation facilities across the UK. 50 participants who currently use opioids and live in homeless hostels in England and Scotland will wear the PneumoWave biosensor for varying periods to collect data over 2,000 participant-days. The biosensor will be linked via Bluetooth to a hub for continuous respiratory data collection. Self-reported drug use during the trial will be measured using drug diaries. Quantitative acceptability data will be measured using structured satisfaction surveys, while qualitative acceptability data will be obtained from interviews and focus groups with both residents and staff. Statistical analysis will include descriptive evaluation of feasibility outcomes, while qualitative data will undergo thematic analysis. The primary objectives of the study are: 1) feasibility of the study protocol within the hostel setting; 2) acceptability and usability of the device among people who use opioids and live in hostels; 3) acceptability of the device among staff who work in hostels and respond to overdose events. Primary outcomes are recruitment, total hours of usable data collected and successful recording of key outcome measures, among others. Trial registration: ISRCTN12060022.

**Results & Significance:**

Findings will inform the feasibility of future integration of chest biosensor technology into hostel settings, assessing participant adherence, usability, and acceptability among people who use substances and staff. Insights gained will support the design of future trials and further development of remote monitoring technologies for overdose prevention and response strategies.

## Introduction

Opioid-related overdose is a major public health crisis, with overdose deaths increasing globally. According to the United Nations Office on Drugs and Crime (UNODC), over 100,000 opioid-related deaths occur worldwide each year, with mortality rates rising due to the increased prevalence of synthetic opioids such as fentanyl [1,2]. In the UK alone, opioid-related deaths have reached record levels [3]. Scotland has experienced particularly high rates, with 1,172 drug-related deaths recorded in 2023, making it one of the highest per capita drug death rates in Europe [4]. People experiencing homelessness tend to have high levels of opioid use and co-existing physical health problems and are at particular risk of overdose [5–8]. Traditional harm reduction approaches, including supervised drug consumption facilities and naloxone distribution, have been introduced as initiatives to reduce overdose mortality [9–11]; however, their reach remains limited due to geographic, legal, and social barriers. Given these limitations, emerging digital health technologies, such as biosensors for real-time respiratory monitoring, present an innovative approach to overdose prevention by providing continuous physiological data that may enable early intervention before fatal respiratory depression occurs.

PneumoWave DC is a wearable biosensor system designed to monitor chest motion. Future versions will aim to detect respiratory depression in individuals at high risk of overdose. The DC system comprises a small chest-worn biosensor that transmits real-time chest motion data to a Hub, the device is designed to be unobtrusive, allowing for prolonged use without discomfort. In future developments the device will be capable of providing real-time alerts when chest motion abnormalities potentially linked to opioid use are detected.

Previous research has demonstrated that wearable biosensors have significant potential for improving health monitoring in various medical contexts, including respiratory diseases such as chronic obstructive pulmonary disease (COPD) and sleep apnoea. Other studies have investigated the use of technologies for opioid-induced respiratory depression, more specifically, studies testing various sensors and devices highlight the potential for wearable real-time monitoring for overdose prevention [12–14]. However, despite these advancements, the practical application of biosensors for harm reduction remains understudied. Most research to date has been conducted in controlled environments such as hospitals or supervised injection sites, leaving a gap in understanding how these devices perform in real-world, community-based settings. This study, RESCU-2, aims to bridge that gap by evaluating the feasibility and acceptability of PneumoWave Biosensor within homeless hostel or supported accommodation environments (equivalent to Housing First in the USA) where individuals may use opioids in private, unsupervised conditions. Supported accommodation facilities provide an opportunity to implement life saving interventions when notified in environments where individuals are already receiving services and support. This study builds upon findings from the RESCU-1 study which explored the feasibility of respiratory monitoring in people recruited in treatment clinics and needle exchanges, and who self-report their drug use.

This study aims to evaluate the feasibility, acceptability, and technical implementation of a wearable biosensor (PneumoWave DC system) to record chest motion in individuals at risk of opioid overdose living in supported accommodation.

The primary objectives are to:

1. Assess the feasibility of implementing the study protocol within supported accommodation settings. This includes recruitment and retention of participants, adherence to study procedures, and overall completeness of data collection.
2. Evaluate the acceptability and usability of the biosensor system among residents who use opioids. This will be informed by participant engagement, qualitative feedback, and satisfaction survey responses.
3. Assess the acceptability of the biosensor system among hostel staff and key stakeholders, particularly those involved in overdose response.
4. Determine the technical feasibility of capturing chest motion data using the PneumoWave DC system in this setting, and evaluate its potential to contribute to overdose detection algorithm development.

Secondary objectives include:

- Descriptive analysis of events where sensor data corresponds to suspected opioid overdose episodes, based on staff reports or participant accounts.
- Evaluation of the system’s ability to detect changes in recorded chest motion during suspected events and to differentiate overdose-related respiratory suppression from typical patterns.
- Exploratory comparison of biosensor traces from overdose and non-overdose periods to assess system sensitivity and specificity in identifying overdose events.

## Methods

### Study design

RESCU-2 is a non-randomised, observational feasibility study designed to assess the implementation of a wearable (PneumoWave DC) biosensor system in supported accommodation facilities. The study combines quantitative and qualitative methods to evaluate feasibility, acceptability, and data capture. It builds on the RESCU-1 study, which explored biosensor use among individuals accessing needle exchange services and self-reporting drug use in community settings. In contrast, RESCU-2 is focused on individuals living in hostels and supported accommodation, including both short- and long-term residents, where unsupervised drug use and overdose risk are common.

### Setting and participants

**Fig 1:**
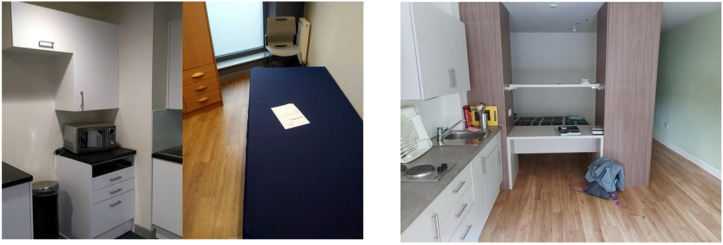
A hostel room in London (left) and Glasgow (right).

The study is conducted at multiple supported accommodation sites across England and Scotland (Fig 1). These organisations provide housing and support services for individuals who experience homelessness or are at risk of drug-related harm, including opioid overdose. The participating sites are selected based on their capacity to facilitate harm reduction interventions and their history of working with individuals with substance use disorders.

There are no exclusions based on length of stay, and both short- and long-term residents are eligible. Participants are enrolled on a rolling basis. Although it must be noted that while the protocol initially includes both short- and long-term residents, current ongoing recruitment has focused solely on longer-term residents, following feedback from participating services that overnight residents are difficult to identify and engage reliably for research participation.

**Fig 2:**
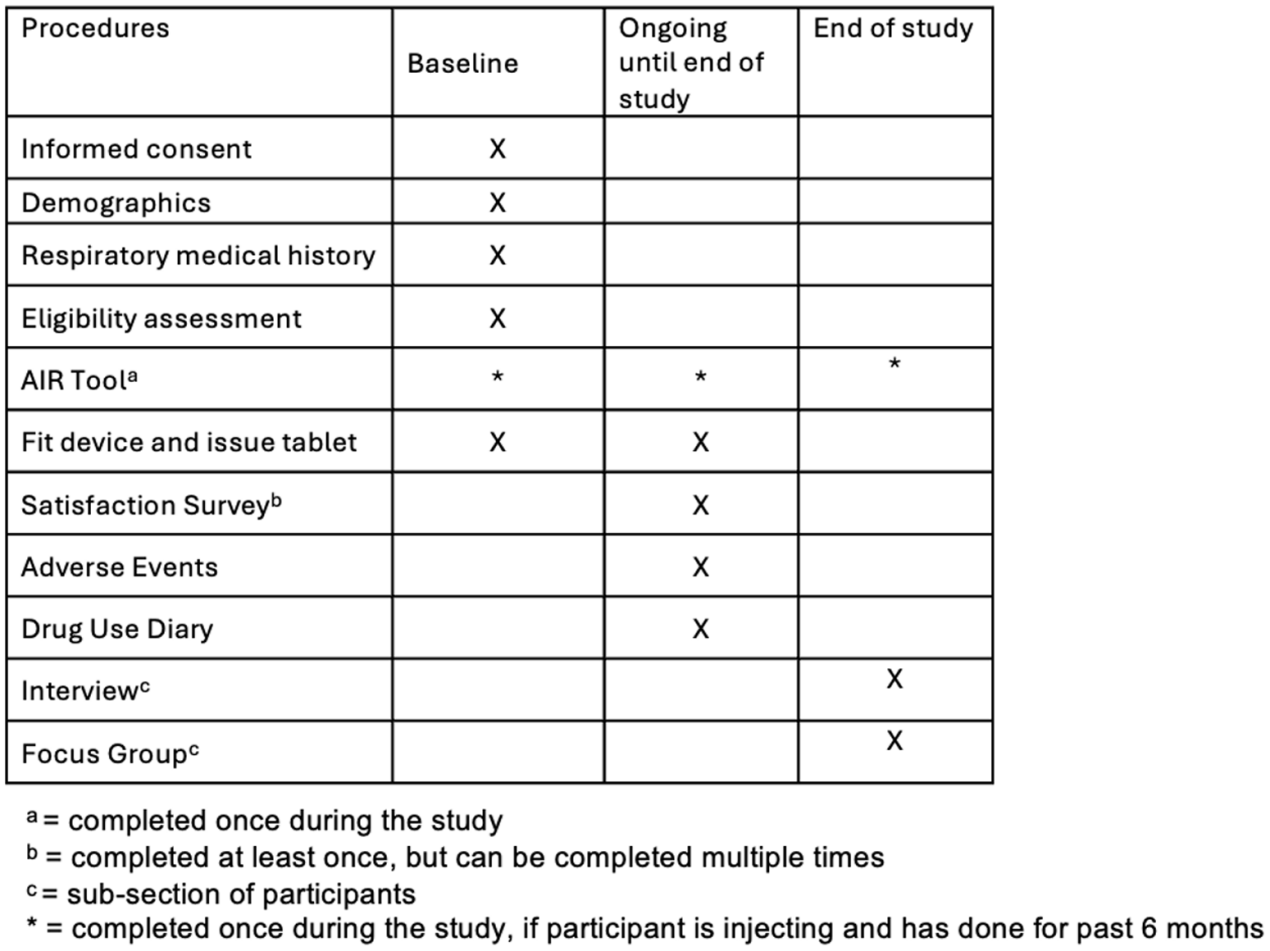
Schedule of procedures. SPIRIT checklist (S1 File) is included as a supplementary file.

Accommodation staff play a key role in participant recruitment and data collection. Staff members will identify eligible individuals based on inclusion criteria and will introduce them to the study during routine interactions, such as welfare checks and scheduled support meetings. Potential participants will receive an overview of the study’s aims and procedures before providing informed consent (Fig 2). Staff will also assist in data collection by documenting participant engagement with the biosensor, noting any relevant observations, and supporting the administration of surveys and qualitative interviews.

50 participants will be recruited over 6–8months to collate 2,000 participant-days of sensor data. Each participant is expected to wear the device for 4 weeks or longer. Recruitment will be conducted on a rolling basis, with eligible participants invited to enrol throughout the study duration.

Staff will maintain a log of recruitment efforts, including reasons for non-participation when applicable, to assess barriers to engagement. This approach ensures that data collection reflects real-world conditions and allows for flexibility in accommodating the needs of a transient and vulnerable population.

### Participant payment

Participants will receive £25 supermarket voucher per week if they show evidence of wearing the sensor and the Hub remains in situ in their residence. Participants will also receive £20 for taking part in a semi-structured interview and £10 for participation in a focus group.

Inclusion criteria:

- Residents of participating supported accommodations
- Aged 18 or above
- Current use of any opioids (e.g., heroin)
- Willingness to wear biosensor
- Ability to provide informed consent

Exclusion criteria:

- Severe cognitive impairment preventing informed consent
- Medical contraindications to biosensor use (e.g., severe dermatological conditions preventing adherence of the device)

### Participant recruitment and enrolment

Potential participants will be identified by accommodation staff and provided with information about the study. Those expressing interest will undergo an informed consent process before enrolment. No formal screening process will be implemented beyond staff assessment and participant self-reporting of opioid use. Consent for satisfaction surveys and interviews will also be sought at the enrolment stage.

### Study procedures

**Fig 3:**
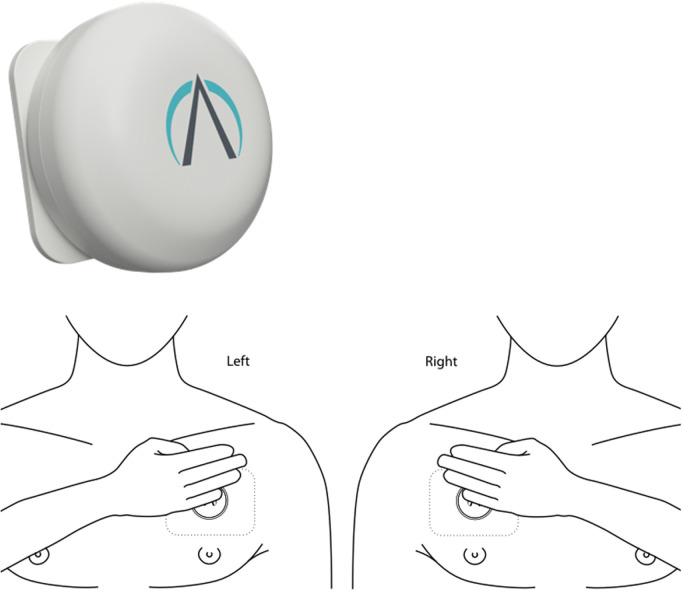
Image of Pneumowave chest sensor and location of placement on the body.

Each participant is issued the PneumoWave DC system (Fig 3), comprising a small, chest-worn biosensor and a local data collection Hub. The device captures motion in three dimensions (x, y, z), which is converted into respiratory rate and waveform data. Data are continuously transmitted to a secure, cloud-based platform via the Hub.

Participants are asked to wear the biosensor as consistently as possible during their stay in the hostel. They are not required to wear it outside the facility. Hostel staff assist with basic maintenance, including support for battery replacement and electrode changes. Staff and participants are provided with brief training and written guidance.

Upon enrolment, participants will receive a wearable biosensor that monitors chest motion and transmits data to a Hub. The study procedures include:

1. Demographics and baseline assessment: collection of age, gender, health status (including respiratory conditions) and opioid use history (abbreviated AIR tool [15,16].
2. Biosensor data: duration worn, number of useable hours recorded, signal quality
3. Drug use diary: participants will self-report drug use via a confidential diary.
4. Overdose reporting: any overdose events observed or reported are documented by hostel staff using a structured form
5. Satisfaction surveys: A brief questionnaire administered to participants to capture perceptions of comfort, usability, and willingness to use the device long term. At least one survey will be completed by each participant to assess usability and comfort, at 4 weeks.
6. Qualitative interviews and focus groups: will be conducted with participants and staff to explore experiences, perceived benefits, and challenges of using the technology.

### Study outcomes

**Fig 4:**
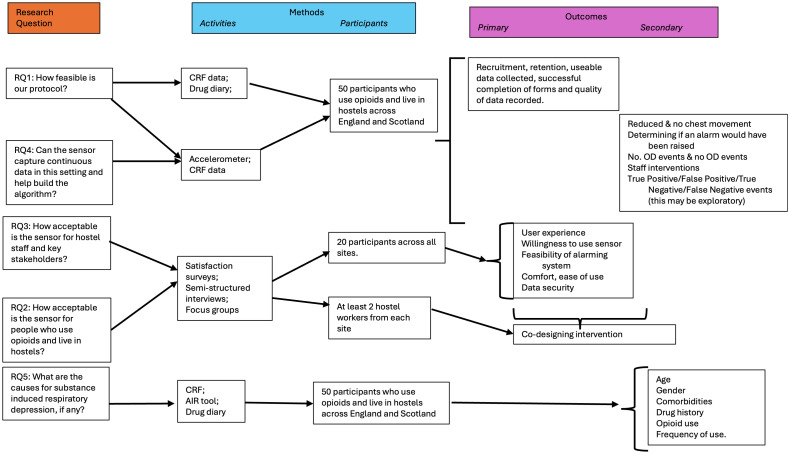
Study Logic Model. Assumptions are: – the study will last 8 months, recruitment will be conducted opportunistically by hostel staff, the sensor will be worn by participants for at least 4 weeks continuously, ideally at all hours, every day.

This study will evaluate both feasibility and exploratory clinical outcomes to determine whether a wearable biosensor (PneumoWave DC) system is suitable for use in supported accommodation settings with people who use opioids (Fig 4).

Primary Outcomes

- Feasibility of protocol delivery, including:
  - Recruitment and retention rates
  - Timeliness and completeness of data collection
  - Estimated time participants wore the biosensor
- Acceptability and usability of the device, based on:
  - Completion of self-reported drug use diaries
  - Responses to participant satisfaction surveys
  - Qualitative feedback from participants and staff
- Technical feasibility of sensor deployment, including:
  - Total hours of usable chest motion data collected
  - Proportion of successful data recordings
  - Operational issues (e.g., battery replacement, electrode adhesion, connectivity)

Secondary outcomes

- Event detection capability, including:
  - Number of events where reduced or absent chest movement was detected
  - Concordance between staff/participant-reported overdose events and sensor data
- Signal quality assessment at the time of suspected overdose events
- Exploratory evaluation of algorithm relevance, including:
  - Qualitative comparison of traces from suspected overdose and non-overdose periods
  - Classification of event detection outcomes (true positive, false positive, false negative)

#### Quantitative measures.

Approximately four weeks after enrolment, a subset of participants and hostel staff are invited to take part in semi-structured interviews. These explore perceptions of the biosensor’s usability, comfort, acceptability, and perceived value. A topic guide will be used and adapted iteratively. Interviews are audio recorded, transcribed, and anonymised for analysis. Where feasible, focus groups are also conducted with staff to explore operational perspectives and overdose response integration.

#### Qualitative interviews.

To explore participant experiences and perceptions of the PneumoWave DC system, semi-structured qualitative interviews will be conducted approximately four weeks after the initiation of data collection. These interviews will provide insights into usability, acceptability, and any challenges encountered while using the biosensor. A structured topic guide will be used to ensure consistency across interviews while allowing for open-ended responses (Supplementary File). This guide will include questions on ease of use, perceived benefits, privacy concerns, and the impact of the biosensor on participants’ daily lives.

Interviews will be conducted face-to-face in a private setting within the supported accommodation facility to ensure confidentiality and participant comfort. Interviews will be audio-recorded with consent from the participant and transcribed verbatim for thematic analysis. Focus groups or interviews with accommodation staff will also be conducted to gather perspectives on the feasibility of implementing the biosensor within supported housing environments. These discussions will explore staff experiences with participant recruitment, device adherence, and any operational challenges that arose during the study.

#### Rationale for study procedures.

Several key decisions were made in designing this study to maximise feasibility and data quality:

- Drug diaries: Self-reported drug use diaries were chosen as they allow for frequent participant input, utilised for matching self-reported substance use recorded chest motion data. This method was also successfully utilised in RESCU-1, making it a logical approach for RESCU-2 to maintain consistency.
- Study setting selection: Supported accommodation settings were chosen due to their role in supporting individuals who might be at risk of overdose. This setting allows for real-world assessment and evaluation of biosensor use, which is essential for its future potential in these settings to prevent fatalities.
- Use of a Hub for data transmission: Instead of relying on participants’ personal devices or providing a smartphone or tablet, the study employs a dedicated Hub to collect and transmit biosensor data. This decision was made in collaboration with accommodation staff for a number of reasons: to reduce technological barriers, ensure reliable data capture across different sites, reduce vulnerability to theft, or risk that the devices could be sold.
  - Participant and Patient Involvement (PPI) and a senior qualitative researcher (PR) informed the topic guide of the study

### Data collection and management

All collected data in the study will be pseudonymised. CRF data will be securely stored on a University of Dundee server. Data entry will be conducted in an encrypted Excel spreadsheet, with de-identified participant codes ensuring confidentiality. Qualitative interviews will be transcribed and anonymised before analysis.

Data from the biosensor will be uploaded securely and automatically to the Hub stored in a small container, located within the hostels. The data storage platform (supplied by Galen Data) complies with Health Insurance Portability and Accountability Act (HIPAA) requirements as set forth in the Galen Cloud HIPAA Compliance Matrix (004–0020), General Data Protection Regulation (GDPR) requirements as set forth in the Galen Cloud GDPR Compliance Matrix (004–0021), and California Consumer Privacy Act (CCPA) requirements. Additionally, Galen Cloud deployed on AWS is HITRUST certified.

A Trial Steering Committee and Data Monitoring Committee will be convened independently from the Sponsor for this study and will meet regularly to monitor the progress of the study and data collection and analysis.

### Ethical and safety considerations

The study is conducted in accordance with Good Clinical Practice (GCP) guidelines and has received ethical approval from the appropriate Research Ethics Committee (REC) from the University of Dundee (UOD-SMED-SLS-Staff-2024-24-107). The Sponsor for the study is University of Dundee.

Written and informed consent will be taken from all participants prior to any study procedures, including both sensor deployment and participation in interviews. Consent is taken by trained research staff or hostel staff and is treated as an ongoing process. Participants have the right to withdraw at any time without consequences. A sample consent form is included as a Supplementary File.

### No minors are involved in the study

Participants experiencing an overdose during the study will receive standard intervention from site staff. An Adverse Events form will be completed and as long as participants continue to fulfil the eligibility criteria they will remain in the study. The biosensor will remain in place during medical interventions when feasible. Ethical safeguards include ensuring that participants understand they can withdraw at any time without penalty.

For qualitative data collection, interviews and focus groups will be audio-recorded with explicit, written informed consent from all participants. Interview recordings will be transcribed verbatim for thematic analysis. Interviews with accommodation staff will also be conducted to gather implementation perspectives, with the same consent procedures applied.

### Statistical and qualitative analysis

The analysis plan is registered on Open Science Framework with the Study Logic model (reference: osf.io/m67nr). Both quantitative and qualitative data will be analysed to assess the feasibility, acceptability, and technical performance of the PneumoWave DC system in supported accommodation settings.

#### Quantitative analysis.

- Descriptive statistics will be used to describe the number of events, evidence of reduced and no chest movement collected by the DC system, participant demographics, self-reported drug use, engagement with the device, and responses to satisfaction surveys.
- Key feasibility indicators, such as device wear time, frequency of use, and reported usability issues, will be examined.
- Measures of variability will be calculated to assess consistency of device use across different participants and sites
- Analyses will explore differences based on demographic factors, prior overdose history, and frequency of opioid use
- If sufficient data are available, exploratory inferential statistics may be conducted to examine relationships between variables.

#### Qualitative analysis.

- Thematic analysis will be conducted on interview and focus group transcripts to identify recurring themes related to feasibility, acceptability, and perceived benefits or barriers to using the biosensor.
- A coding framework will be developed iteratively, with transcripts independently reviewed by multiple researchers to ensure consistency.
- Attention will be given to both participant and staff perspectives, highlighting differences in experiences and expectations regarding the technology.
- Data saturation will be assessed to ensure that key themes are adequately represented across participant responses.

### Quantitative analysis

For the hostel outcome measures (e.g., demographic data) and satisfaction survey, descriptive statistics will be reported using means and standard deviation for continuous data. Absolute numbers and percentages will be reported for categorical data. Any statistical software can be used.

Key feasibility indicators include:

- Number of participants enrolled and retained
- Total hours of usable biosensor data recorded
- Percentage of completed self-reported drug use entries
- Frequency and nature of technical issues (e.g., battery replacement, electrode use)
- Timeliness and completeness of overdose reporting from staff

### Biosensor analyses

#### Reference event.

Evidence of reduced and no chest movement within the sensor data will be compared to the observed and recorded reference events. Reference events will comprise of staff observed events and reports and drug use diary records. Staff-identified events are any event that has required an intervention by staff in the hostel. Interventions can range from a simple audio stimulus, up to and including requesting an ambulance to attend. These events include those where overdose may not have occurred, but the response will have been managed in the same way according to the site’s incident response. These incidences will be recorded as an ‘acute event’ or ‘prompt event’. Where an overdose has been clearly identified using the tools mentioned above, these incidences will be identified as ‘overdose event.’ Analysis will incorporate both types of ‘reference’ events. Biosensor data will be analysed over a period of 30 minutes prior to each reference event. We expect the following possible scenarios to occur: 1) no opioid use reported, overdose event; 2) opioid use reported but no overdose event; 3) opioid use and overdose event.

#### Algorithm under test.

The PneumoWave Hub transmits Biosensor data to a Cloud Storage facility. The stored data is then processed using experimental algorithms for the presence of reduced chest movement events.

The performance of the Algorithm (Under Test, AUT) will be compared to the reference events at the hostels in terms of the AUT’s sensitivity and false alarm rate, if this is possible. If the target numbers for sample size and acute events are not reached, analysis will still be possible and feasible, but it may be exploratory.

#### Qualitative analyses.

Focus group and interview audio recordings will be transcribed verbatim. Transcripts will be analysed and a coding framework will be developed using thematic analysis, specifically an inductive thematic approach (Braun & Clarke, 2006). Thematic analysis will help identify explicit meanings arising from the interviews and enable the interpretation of underlying, implicit meanings. This approach will provide insights into participants’ attitudes, feelings, and perceptions regarding the acceptability of wearing the device, and support the identification of suitable strategies for developing a clinical intervention pathway.

Multiple researchers will review transcripts to ensure coding reliability and identify recurring themes related to:

- Participant experiences of wearing the biosensor
- Perceived barriers and facilitators
- Acceptability in day-to-day use
- Staff perspectives on device integration into overdose response workflows

NVivo or equivalent software will be used to support coding and data organisation. Emerging themes will be compared across stakeholder groups (residents vs staff) to explore shared and divergent perspectives. Qualitative findings will be integrated with quantitative results to provide a comprehensive assessment of feasibility and acceptability.

### Protocol amendments and early termination

Any significant amendments to the study protocol will be reviewed and approved by the REC and the study sponsor. Participants who lose capacity will be withdrawn from the study to ensure ethical compliance. The study may be terminated early due to safety concerns, funding constraints, or administrative decisions, with appropriate reporting to regulatory bodies.

### Status and timeline

This study is part of a 12-month funded study. Funding started in September 2024, when approvals were sought and sites were set up from September to December 2024. The first participant was recruited on 28 January 2025 and is ongoing. Recruitment and data collection will continue until 31 July 2025. Data analysis and results have not yet been generated. Data analysis and results are expected in August 2025 with the final report expect to be completed by 31 August 2025, with dissemination following thereafter.

Although the original protocol included the possibility of enrolling short-term or overnight residents, and this is described in the manuscript, early consultation with participating services indicated that identifying and engaging this group in a meaningful way would not be feasible. As a result, recruitment has focused exclusively on longer-term residents, who are more stable and accessible for follow-up and data collection.

## Discussion

Opioid overdose continues to be a leading cause of drug-related mortality worldwide, with increasing concerns about its impact on vulnerable populations. Traditional harm reduction strategies, including supervised consumption facilities and naloxone distribution, are effective for some but remain limited in their reach and accessibility. Wearable biosensors represent an emerging technological solution to this crisis, offering real-time physiological monitoring that may facilitate early intervention and overdose prevention. This study examines the feasibility and acceptability of the PneumoWave biosensor to monitor chest motion in supported accommodation settings, contributing to the growing body of research exploring digital health interventions for substance use disorders.

### Limitations of study design

As an observational study without a control group, this research does not allow for causal inferences regarding the effectiveness of the PneumoWave DC system in preventing overdose-related fatalities. Additionally, participant adherence may vary, potentially affecting data consistency. The study is conducted within supported accommodation facilities, which will not fully represent the broader community of individuals at risk of opioid overdose. Variability in participant engagement and technical challenges related to biosensor use may also introduce potential biases.

### Comparison with similar work

Previous studies, such as those conducted in medically supervised injecting centres [17], have assessed the feasibility of wearable biosensors for opioid overdose detection. The ODSEEN study, for example, has examined the PneumoWave system in a supervised drug consumption setting, yielding promising results regarding its ability to utilise recorded chest motion to develop an application to detect respiratory depression. Another study monitored people who had used opioids in a women’s shelter in the USA using continuous pulse oximetry [18].

However, the current study expands upon previous work by evaluating the device in supported accommodation settings where supervision is less structured, reflecting real-world usage conditions more accurately. Unlike prior research focusing on short-term biosensor use during single drug consumption events, RESCU-2 explores long-term feasibility, adherence, and acceptability among individuals in semi-independent living environments.

Findings from this study will be compared with previous research on wearable biosensors for opioid overdose prevention, including RESCU-1 which is currently in preparation and evaluates the same chest sensor, in an earlier prototype form, among participants who wear it at home while receiving OST. Additionally, comparisons will be drawn with broader studies examining the feasibility and acceptability of wearable devices designed to address the overdose crisis [12,19–23]. The effectiveness and user acceptability of these technologies have shown considerable variation, reflecting differences in study populations, device designs, and monitoring settings. While there is a growing body of research in this field, most studies focus on technologies that are still in early development or have yet to be widely implemented in real-world harm reduction settings [24]. Currently, there is no published work evaluating a remote chest sensor capable of reliably detecting chest wall movement in the context of opioid overdose, highlighting the unique contribution of this study to the existing literature.

### Strengths and weaknesses

A major strength of this study is its focus on real-world implementation of the PneumoWave DC system in a setting where overdose risk is high, but immediate medical supervision is not always available. The inclusion of both quantitative and qualitative data enhances the comprehensiveness of the findings, allowing for a deeper understanding of both technical feasibility and useability. By capturing structured metrics and nuanced user feedback, the study will generate findings that are directly relevant to service providers, researchers and policymakers.

Limitations include the reliance on self-reported drug use data, which may be influenced by recall bias or social desirability effects. Drug use diaries, essential for accurately correlating chest motion data with drug administration, may be incomplete or lack sufficient detail. While qualitative insights offer valuable context, subjective responses may not always align with objective biosensor data. Additionally, practical and technical factors, such as device connectivity disruptions and data transmission variability may affect the completeness of the database.

### Dissemination plans

Findings from this study will be disseminated through peer-reviewed journal publications, conference presentations, and community reports tailored for stakeholders. Participants who remain in supported accommodation at the time of publication will receive a summary of results. Insights will also be shared with policymakers to inform future initiatives and potential integration of biosensor technology into public health strategies.

## Conclusion

The RESCU-2 study aims to provide crucial insights into the feasibility and acceptability of wearable biosensors for overdose detection in supported accommodation settings. By assessing both technical performance and insights on facilitators and barriers on use of the technology, this research will help determine the potential for integrating real-time respiratory monitoring into relevant settings. Findings from this study will inform future interventions and policy decisions aimed at reducing opioid-related mortality in high-risk populations.

## Supporting information

S1 File SPIRIT checklist. (DOCX)

S2 File Human Subjects Checklist. (DOCX)

## References

[1] Ahmad SP, Rossen LM. Provisional drug overdose death counts. https://www.cdc.gov/nchs/nvss/vsrr/drug-overdose-data.htm

[2] Ahmad FB, Cisewski JA, Rossen LM, Sutton P. Provisional drug overdose death counts. https://www.cdc.gov/nchs/nvss/vsrr/drug-overdose-data.htm. 2023.

[3] Wells C. Deaths related to drug poisoning in England and Wales, 2008. Health Stat Q. 2009;(43):48–55. doi: 10.1057/hsq.2009.27 19774834

[4] National Records of Scotland. Drug-related deaths in Scotland in 2023. 2024. https://www.nrscotland.gov.uk/publications/drug-related-deaths-in-scotland-in-2023/

[5] Poe B. Addressing the Opioid Epidemic. How the Opioid Crisis Affects Homeless Populations. National Health Care for the Homeless Council. 2017. www.nhchc.org

[6] Lowrie R, McPherson A, Mair FS, Stock K, Jones C, Maguire D, et al. Baseline characteristics of people experiencing homelessness with a recent drug overdose in the PHOENIx pilot randomised controlled trial. Harm Reduct J. 2023;20(1):46. doi: 10.1186/s12954-023-00771-4 37016418 PMC10071267

[7] Campbell A, Millen S, Guo L, Jordan U, Taylor-Beswick A, Rintoul C, et al. Reducing opioid related deaths for individuals who are at high risk of death from overdose: a co-production study with people housed within prison and hostel accommodation during Covid-19. Front Public Health. 2023;11:1080629. doi: 10.3389/fpubh.2023.1080629 37404271 PMC10316785

[8] Fine DR, Dickins KA, Adams LD, De Las Nueces D, Weinstock K, Wright J, et al. Drug Overdose Mortality Among People Experiencing Homelessness, 2003 to 2018. JAMA Netw Open. 2022;5(1):e2142676. doi: 10.1001/jamanetworkopen.2021.42676 34994792 PMC8742197

[9] Fischer LS, Asher A, Stein R, Becasen J, Doreson A, Mermin J, et al. Effectiveness of naloxone distribution in community settings to reduce opioid overdose deaths among people who use drugs: a systematic review and meta-analysis. BMC Public Health. 2025;25(1):1135. doi: 10.1186/s12889-025-22210-8 40133970 PMC11934755

[10] Naumann RB, Durrance CP, Ranapurwala SI, Austin AE, Proescholdbell S, Childs R, et al. Impact of a community-based naloxone distribution program on opioid overdose death rates. Drug Alcohol Depend. 2019;204:107536. doi: 10.1016/j.drugalcdep.2019.06.038 31494440 PMC8107918

[11] Livingston E. UK to open its first safe drug consumption room amid soaring deaths. The Observer. 2024.

[12] Nandakumar R, Gollakota S, Sunshine JE. Opioid overdose detection using smartphones. Sci Transl Med. 2019;11(474):eaau8914. doi: 10.1126/scitranslmed.aau8914 30626717

[13] Goldfine C, Lai JT, Lucey E, Newcomb M, Carreiro S. Wearable and Wireless mHealth Technologies for Substance Use Disorder. Curr Addict Rep. 2020;7(3):291–300. doi: 10.1007/s40429-020-00318-8 33738178 PMC7963000

[14] Carreiro S, Wittbold K, Indic P, Fang H, Zhang J, Boyer EW. Wearable Biosensors to Detect Physiologic Change During Opioid Use. J Med Toxicol. 2016;12(3):255–62. doi: 10.1007/s13181-016-0557-5 27334894 PMC4996791

[15] NEO Development Group – subgroup of the Prevention Leads Group. NEO Development Group, Blood Borne Virus (Non-Sexual Transmission) Prevention Leads. 2025. Accessed: Apr. 09, 2025 https://neo360.co/.

[16] Neo 360. Neo360: Empowering harm reduction through data. Neo360. 2025 April 9.

[17] Tas B, Lawn W, Jauncey M, Bartlett M, Dietze P, O’Keefe D, et al. Overdose Detection Among High-Risk Opioid Users Via a Wearable Chest Sensor in a Supervised Injecting Facility: Protocol for an Observational Study. JMIR Res Protoc. 2024;13:e57367. doi: 10.2196/57367 39255471 PMC11422748

[18] Beaugard CA, Khudairi F, Yesufu O, Farina A, Laks J. “I don’t think of it as a shelter. I say I’m going home”: a qualitative evaluation of a low-threshold shelter for women who use drugs. Harm Reduct J. 2024;21(1):44. doi: 10.1186/s12954-024-00930-1 38374063 PMC10877776

[19] Tas B, Walker H, Lawn W, Matcham F, Traykova EV, Evans RAS, et al. What impacts the acceptability of wearable devices that detect opioid overdose in people who use opioids? A qualitative study. Drug Alcohol Rev. 2024;43(1):213–25. doi: 10.1111/dar.13737 37596977

[20] Kanter K, Gallagher R, Eweje F, Lee A, Gordon D, Landy S, et al. Willingness to use a wearable device capable of detecting and reversing overdose among people who use opioids in Philadelphia. Harm Reduct J. 2021;18(1). doi: 10.1186/s12954-021-00522-3 PMC8299455 34301246

[21] Ahamad K, Dong H, Johnson C, Hyashi K, DeBeck K, Milloy MJ, et al. Factors associated with willingness to wear an electronic overdose detection device. Addict Sci Clin Pract. 2019;14(1):23. doi: 10.1186/s13722-019-0153-5 31269963 PMC6610816

[22] Dhowan B, Lim J, MacLean MD, Berman AG, Kim MK, Yang Q, et al. Simple minimally-invasive automatic antidote delivery device (A2D2) towards closed-loop reversal of opioid overdose. J Control Release. 2019;306:130–7. doi: 10.1016/j.jconrel.2019.05.041 31158402 PMC6629496

[23] Imtiaz MS, Bandoian CV, Santoro TJ. Hypoxia driven opioid targeted automated device for overdose rescue. Sci Rep. 2021;11(1):24513. doi: 10.1038/s41598-021-04094-x 34972818 PMC8720093

[24] Tas B, Lawn W, Traykova EV, Evans RAS, Murvai B, Walker H, et al. A scoping review of mHealth technologies for opioid overdose prevention, detection and response. Drug Alcohol Rev. 2023;42(4):748–64. doi: 10.1111/dar.13645 36933892

